# PReS-FINAL-2092: Bone marrow cells (BMC) added to platelet-rich plasma (PRP) for treatment of bone degenerative processes in JIA patients: follow-up of 2 cases

**DOI:** 10.1186/1546-0096-11-S2-P104

**Published:** 2013-12-05

**Authors:** P Salvati, S Callegari, G Tripodi, MB Michelis, S Boero, R Lorini, MG Alpigiani

**Affiliations:** 1 Pediatrics, Istituto G. Gaslini, Genova, Italy; 2 Istituto G. Gaslini, Genova, Italy; 3 Service of Immuno-Hematology and Transfusion Medicine, Instituto G. Gaslini, Genova, Italy; 4 Pediatric Surgery, Istituto G. Gaslini, Genova, Italy

## Introduction

In Regenerative Medicine one or more regenerative factors can be applied inside a cartilagine or bone defect to obtain a more rapid and complete healing. Bone Marrow Cells (BMC) added to Platelet-Rich Plasma (PRP) contain stromal cells which can differentiate in osteoclasts and osteoblasts and can be able to form osteogenic tissue and to repair bone defects secondary to degenerative processes.

## Objectives

We report 2 cases in which we implanted BMC plus PRP in the osteonecrotic region with a clinical and imaging follow-up.

## Methods

The first case is a 17-year old boy, followed at our Department, affected by JIA since he was 2 years old. He presented a systemic form, evolved into a polyarticular form, treated with steroids and immunosuppressor drugs. The patient had a good response to treatment with Enbrel, which he is still taking. In January 2009, he presented right hip pain and functional limitation. In July 2009 he underwent MRI of the hip joints which showed osteonecrosis in chondral/subchondral regions at the superior-external convexity of the right femoral head. We recommended deambulation with crutches and no weight bearing. Because of the persistence of joint symptoms, in July 2010 we implanted BMC plus PRP in the osteonecrotic region with improvement of pain and mobilization. In October 2010, he presented left hip pain. MRI showed focal osteonecrosis in subchodral region of left femoral head convexity. For this reason, we made a second BMC plus PRP implantation in the left hip. The MRI, made on January 2012, showed any changes concerning the morphology of femoral heads and subchondral erosions. The last MRI, made on December 2012 showed no changing of lesions and a mild improvement of right hip. Our patient autonomous walks, without joint pain and with improved hip movements, since January 2011; he keeps on Enbrel and nsaids. The second case is a 20-year and 4 month old girl with extended oligoarticular JIA diagnosed when she was 2 years old. On July 2012 after an injury, a flare occurred in the left knee. MRI, made on October 2012 showed intra-articular effusion with diffuse synovial thickening, small bone subchondral erosions of medial femoral condyle. She underwent a surgical procedure where we implantated BMC plus PRP on January 2013 which resulted in improvements of mobility and reduction of pain.

## Results

All two patients had an improvement of their mobility and pain reduction with no progression of articular damages on MRI imaging.

## Conclusion

To the best of our knowledge, there are no literature data on the use of BMC plus PRP in pediatric patients affected by JIA. Considering the obvious limitations of our case reports, we observed a good short-term outcome. Therefore, follow-up is essential to check if BMC plus PRP implantation represents only a palliative care to delay surgical treatment or if it is a valid alternative to traditional orthopedic surgery.

## Disclosure of interest

None declared.
